# Stimulated echo acquisition mode (STEAM) diffusion tensor imaging with different diffusion encoding times in the supraspinatus muscle: Test–retest reliability and comparison to spin echo diffusion tensor imaging

**DOI:** 10.1002/nbm.5279

**Published:** 2024-10-24

**Authors:** Adrian Alexander Marth, Stefan Sommer, Thorsten Feiweier, Reto Sutter, Daniel Nanz, Constantin von Deuster

**Affiliations:** ^1^ Swiss Center for Musculoskeletal Imaging (SCMI) Balgrist Campus AG Zurich Switzerland; ^2^ Department of Radiology Balgrist University Hospital Zurich Switzerland; ^3^ Advanced Clinical Imaging Technology Siemens Healthineers International AG Zurich Switzerland; ^4^ Siemens Healthineers AG Erlangen Germany; ^5^ Medical Faculty University of Zurich (UZH) Zurich Switzerland

**Keywords:** diffusion tensor imaging, muscle, stimulated Echo

## Abstract

Diffusion tensor imaging (DTI) provides insight into the skeletal muscle microstructure and can be acquired using a stimulated echo acquisition mode (STEAM)‐based approach to quantify time‐dependent tissue diffusion. This study examined diffusion metrics and signal‐to‐noise ratio (SNR) in the supraspinatus muscle obtained with a STEAM‐DTI sequence with different diffusion encoding times (Δ) and compared them to measures from a spin echo (SE) sequence. Ten healthy subjects (mean age 31.5 ± 4.7 years; five females) underwent 3‐Tesla STEAM and SE‐DTI of the shoulder in three sessions. STEAM was acquired with Δ of 100/200/400/600 ms. The diffusion encoding time in SE scans was 19 ms (*b* = 500 s/mm^2^). Region of interest‐based measurement of fractional anisotropy (FA), mean diffusivity (MD), and SNR was performed. Intraclass correlation coefficients (ICCs) were computed to assess test–retest reliability. ANOVA with post‐hoc pairwise tests was used to compare measures between different Δ of STEAM as well as STEAM and SE, respectively. FA was significantly higher (FA_STEAM_: 0.38–0.46 vs. FA_SE_: 0.26) and MD significantly lower (MD_STEAM_: 1.20–1.33 vs. MD_SE_: 1.62 × 10^−3^ mm^2^/s) in STEAM compared to SE (*p* < 0.001, respectively). SNR was significantly higher for SE (72.3 ± 8.7) than for STEAM (*p* < 0.001). ICCs were excellent for FA in STEAM (≥0.911) and SE (0.960). For MD, ICCs were good for STEAM_100ms–600ms_ (≥0.759) and SE (0.752). STEAM and SE exhibited excellent reliability for FA and good reliability for MD in the supraspinatus muscle. SNR was significantly higher in SE compared to STEAM.

## Graphical abstract

This study evaluated diffusion metrics (fractional anisotropy [FA], mean diffusivity [MD]) and signal‐to‐noise ratio (SNR) in the supraspinatus muscle obtained with a stimulated echo acquisition mode (STEAM) diffusion tensor imaging (DTI) sequence with different mixing times and compared them to measurements obtained with a spin echo (SE) sequence. STEAM resulted in higher FA and lower MD compared to SE. SNR decreased with increasing STEAM mixing time. Test–retest reliability for diffusion metrics was good to excellent for both STEAM and SE.

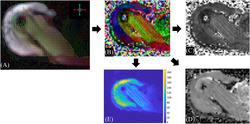

**Abbreviations**
DTI: Diffusion tensor imaging
FA: Fractional anisotropy
MD: Mean diffusivity
SE: Spin echo
SNR: Signal‐to‐noise ratio
STEAM: Stimulated echo acquisition mode

## 1 INTRODUCTION

Diffusion tensor imaging (DTI) has gained recognition for providing insight into muscle microstructure by assessing restricted water diffusion and characterizing muscle anatomy.^1^, ^2^ Diffusion parameters such as anisotropy (fractional anisotropy, FA) and mean diffusivity (MD) have been employed to assess and monitor pathological conditions affecting the structural integrity of muscles, as observed in muscular dystrophy.^3^ Furthermore, it is possible to visualize and quantify exercise‐induced changes following sports activities^4^ and tissue alterations due to injuries,^5^ which cannot be detected by conventional imaging.^6^

Technical challenges in muscle DTI include eddy‐current induced image distortion, low signal‐to‐noise ratio (SNR) due to T2 decay, and an inherent sensitivity to off‐resonances.^7^, ^8^, ^9^ Furthermore, conventional spin echo (SE) DTI sequences with long diffusion encoding times are nonfavorable for muscle imaging due to a prolonged TE (T2 of muscle: ~30–50 ms at 3 Tesla).^10^, ^11^ To increase diffusion encoding time (Δ) and hence enhance diffusion sensitivity, a stimulated echo acquisition mode (STEAM) DTI sequence has been introduced as an alternative to SE sequences. In STEAM DTI, the diffusion encoding time can be modified by varying the time between the second and the third radiofrequency pulses, referred to as “mixing time”. Given the relatively moderate diffusion encoding gradient strength and duration for the same *b*‐value, STEAM has a lower sensitivity to T2 relaxation^12^, ^13^ and eddy currents compared to SE^7^, ^14^ and has been used to assess the skeletal muscle with a focus on the lower extremity.^7^, ^8^, ^12^, ^15^, ^16^, ^17^, ^18^, ^19^, ^20^, ^21^ However, no study has yet attempted to transfer current knowledge from the lower extremity to the shoulder girdle, which is challenging for a variety of technical factors, including field inhomogeneities and susceptibility to motion artifacts.

Therefore, the aim of this study was to extend existing knowledge on the feasibility of STEAM by evaluating the test–retest reliability of DTI metrics (FA and MD) and assessing SNR of STEAM DTI at different Δ in the supraspinatus (SSP) muscle, along with a comparison of results with a conventional SE DTI protocol.

## 2 MATERIAL AND METHODS

### 2.1 Participants

In this prospective study approved by the local institutional review board (Cantonal Ethics Committee, Zurich, Switzerland), 10 healthy volunteers with no prior history of muscle or shoulder injury, muscle disease, or previous shoulder surgery participated after providing written informed consent. The left or right shoulder was selected at random prior to undergoing MRI. Eligibility for this study also required the absence of signs of fever at each examination time point. All procedures were conducted in compliance with the ethical standards set by the institutional and/or national research committee, as well as in accordance with the 1964 Helsinki Declaration and its subsequent amendments.

### 2.2 Imaging protocol

MR images were acquired on a 3‐T system (MAGNETOM Prisma, Siemens Healthineers, Erlangen, Germany) with a maximum gradient strength of 80 mT/m and a maximum slew rate of 200 T/ms using a dedicated 16‐channel shoulder coil. Between May and August 2023, three MRI exams per volunteer were performed at 1‐week intervals, resulting in a total of 30 MRI exams. The MRI examinations were conducted following a 30‐min acclimatization period in the waiting room of the facility at a room temperature of 22.0°C. All examinations were performed in the afternoon hours, and women's appointments were scheduled to the post‐ovulatory phase to avoid inherent differences in body temperature between the sexes. Patient positioning and immobilization were performed according to the clinical standard. Diffusion along 64 directions was encoded^22^, ^23^ with three “*b* = 0” s/mm^2^ images as reference utilizing a monopolar STEAM research sequence (Δ:100/200/400/600 ms) as well as a monopolar SE‐DTI sequence with a single‐shot echo planar imaging (EPI) readout. Fat saturation was achieved by fat‐selective excitation followed by signal dephasing preparation module. In the UI, the “Strong” mode was selected.

The axial plane of the DTI sequences was positioned along the intramuscular part of the SSP tendon for all scans for optimum visualization of the muscle and to ensure geometric alignment between different examination time points. The DTI sequences were followed by a T1‐weighted sequence for anatomical reference. Total scan duration was 45:49 min. Relevant sequence parameters are summarized in Table 1. During the study period, all participants were instructed not to engage in any sports activities or physical work involving the shoulder girdle. Qualitative image readout to exclude pathologies or other incidental findings was performed on T1‐weighted images by a fellowship‐trained radiologist with 5 years of experience in musculoskeletal radiology (A. A. M.).

**TABLE 1:** Sequence parameters of the shoulder MR imaging protocol.

	T1w	SE‐DTI	STEAM‐DTI
TR (ms)	700	6700	6700/6700/6700/9700
TE (ms)	11	42	36
Δ (ms)	‐	‐	100/200/400/600
Slice thickness (mm)	3	3	3
Number of slices	23	15	15
Acquisition matrix size	576 × 576	64 × 64	64 × 64
FoV (mm^2^)	192 × 192	192 × 192	192 × 192
Phase oversampling (%)	100	0	0
Phase encoding direction	A‐P	A‐P	A‐P
Acceleration factor	2	2	2
Diffusion directions	‐	64	64
*b*‐values for “*b* = 0 s/mm^2^” (s/mm^2^)^a^	‐	0	12/22/46/69
*b*‐values for “*b* = 500 s/mm^2^” (s/mm^2^)^b^	‐	500	499–501
Receive bandwidth (Hz/pixel)	‐230	2005	2005
Partial Fourier encoding	Off	7/8	7/8
Averages/*b*‐value	‐	1	1
Acquisition time (min:sec)	03:12	07:51	07:49/07:49/07:49/11:19

Abbreviations: A‐P, anterior to posterior phase encoding direction; DTI, diffusion tensor imaging; FoV, field of view; SE, spin echo; STEAM, stimulated echo acquisition mode; TE, echo time; Δ, diffusion encoding time; TR, repetition time.

^a^ Nominal *b*‐value: 0 s/mm^2^: In case of STEAM, effective *b*‐values for Δ = 100/200/400/600 ms.

^b^ Nominal *b*‐value: 500 s/mm^2^: In case of STEAM, effective *b*‐value range for all directions and Δs.

### 2.3 Image postprocessing

Prior to diffusion tensor fitting, images were examined for potential signal voids and geometric inconsistencies due to eddy‐current induced distortions or physiological motion, followed by nonrigid image registration correction.^24^ To this end, all other images were registered to the first *“b* = 0” s/mm^2^ image. Diffusion tensors were computed with a custom‐made Matlab tool “DTIgui” (R2020b, Mathworks, Natick, MA, USA). Cross term effects (e.g., due to slice select, dephasing, and imaging gradients) were considered during b‐Matrix calculation.^25^

### 2.4 Quantitative image analysis

**FIGURE 1:**
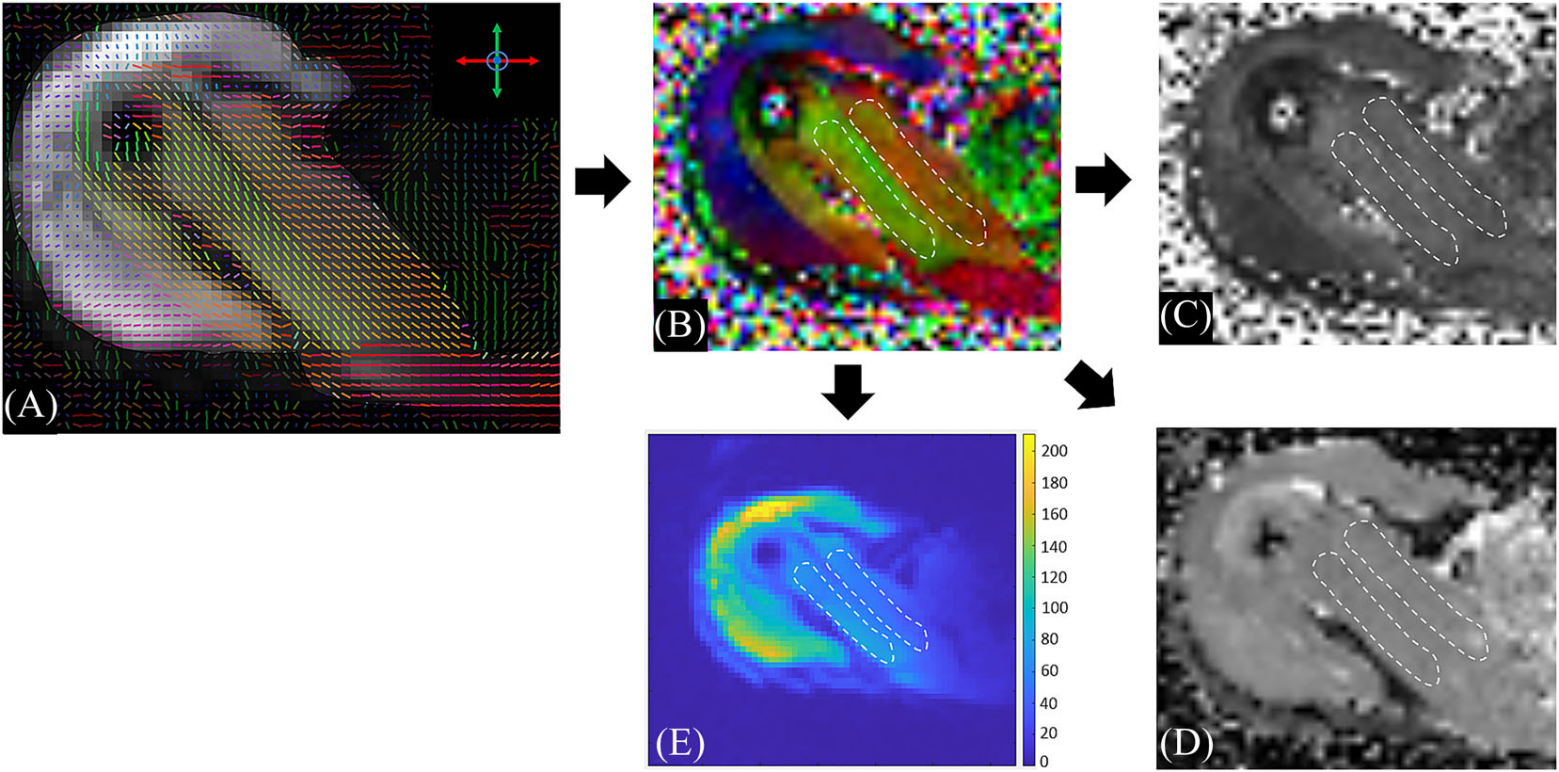
Color‐coded directions of the largest eigenvector in each voxel are shown in (A). Freehand ROIs were drawn in anterior and posterior fiber bundles (dashed areas) of the supraspinatus muscle in corresponding color‐coded FA maps (B). ROIs were then copied to the greyscale FA and MD maps to obtain diffusion metrics (C, D) and to the subtraction map of two *b* = 0 s/mm^2^ to obtain signal‐to‐noise ratio (E). All images are shown in the axial plane for the right shoulder in a 31‐year‐old healthy male subject. FA, fractional anisotropy; MD, mean diffusivity; ROI, regions of interest.

Images were viewed on a dedicated picture archiving and communications system (PACS) work station by a fellowship‐trained radiologist (A. A. M.), who was blinded to participant data. After assessment of the anatomical sequence to exclude potential pathologies and to gain anatomical confidence, 2D regions of interest (ROIs) were drawn on a single slice in the color‐coded FA maps using ITK‐SNAP (v4.0.2).^26^ These freehand ROIs included the anterior and posterior portions at the maximum extent of the anterior SSP muscle bundle, respectively, while carefully excluding the SSP tendon and surrounding structures (Figure 1). In a next step, ROIs were copied to the previously generated greyscale FA and MD maps. SNR was calculated within 10 mm^2^ ROIs in the anterior bundle of the SSP by measuring the mean muscle signal in the *b* = 0 s/mm^2^ images divided by the standard deviation of noise which was estimated by pairwise subtraction of *b* = 0 s/mm^2^ images as described by Froeling et al.^27^

### 2.5 Statistical analysis

All statistical analyses were performed in SPSS (v29, IBM Corp., Armonk, USA). The Shapiro–Wilk test was used to assess normal distribution of continuous data. If normally distributed, data are presented as mean ± standard deviation, and in cases of non‐normal distribution as median with interquartile range in parentheses. For test–retest reliability of quantitative measurements, intraclass correlation coefficients (ICCs) using a two‐way mixed model were calculated. Results were categorized according to Koo and Li^28^: ICC > 0.90: excellent; 0.75–0.90 = good; 0.50–0.75 = moderate; < 0.50 = poor. Mean bias was calculated as the mean difference between FA and MD values at different examination sessions. Measurements between different Δ of STEAM were compared using Friedman's two‐way analysis of variance with pairwise post‐hoc tests. *P‐*values were corrected for multiple comparisons using the Bonferroni procedure. For comparison of STEAM and SE measurements, the Wilcoxon signed rank test was used. *P*‐values below an alpha level of 0.05 were considered significant.

## 3 RESULTS

### 3.1 Participants

**FIGURE 2:**
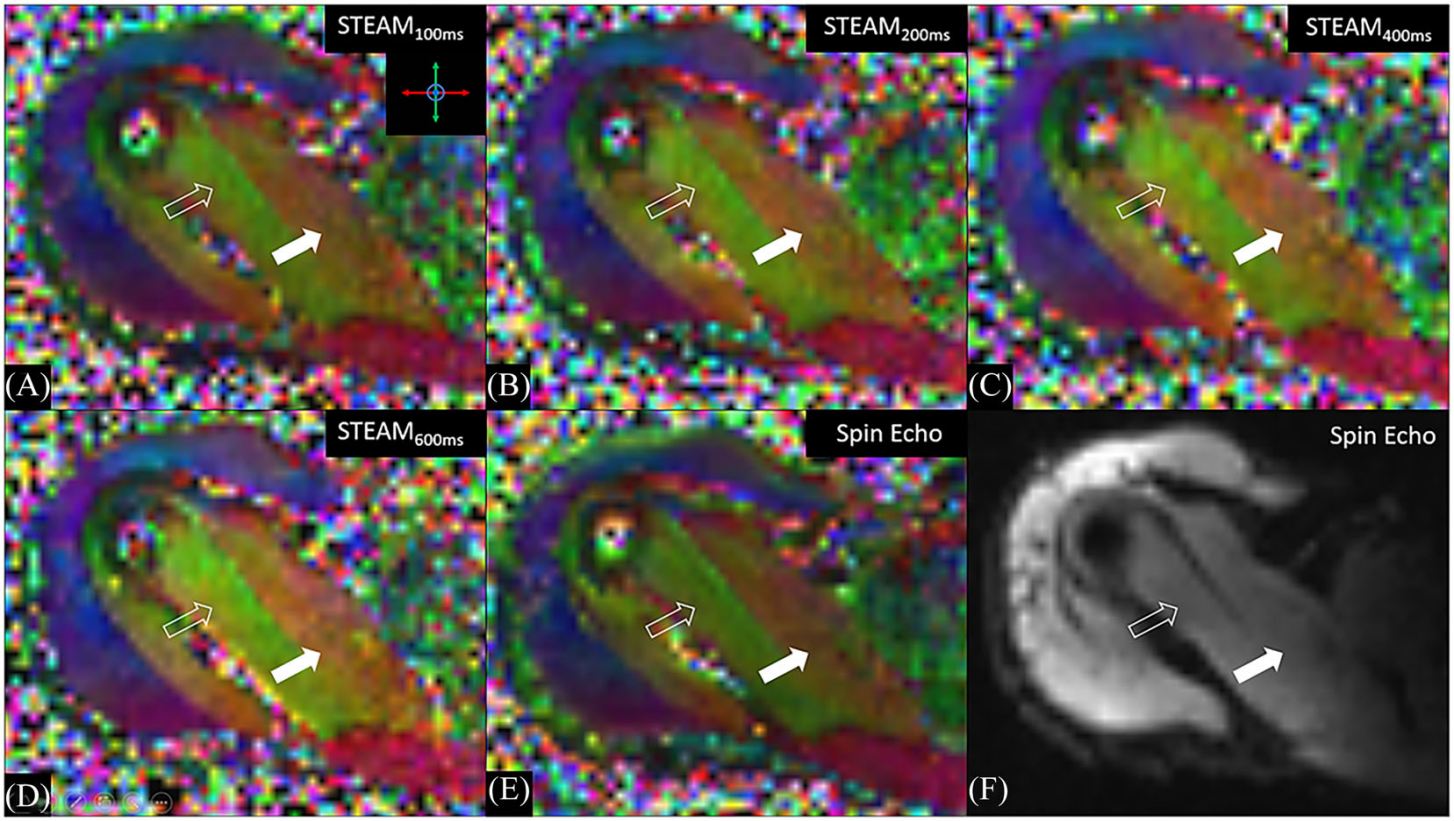
DTI‐derived color‐coded fractional anisotropy maps (A–E) and trace image (F) of different diffusion tensor imaging sequences acquired in the axial plane in the right shoulder of a 31‐year‐old healthy male subject. Colors indicate the principal direction of diffusion (the largest eigenvector) as shown in the coordinate system. Note the excellent delineation of the SSP bundles anterior (solid arrows) and posterior (outline arrows) to the tendon. DTI, diffusion tensor imaging; SSP supraspinatus muscle; STEAM, stimulated echo acquisition mode.

Mean age of the 10 healthy volunteers (five females, five males) was 32.0 ± 4.7 years with a mean body mass index of 23.0 ± 2.6 kg/m^2^. The left shoulder was examined in four cases and the right shoulder in six cases. No volunteers were excluded due to signs of illness (e.g., fever). The qualitative image analysis of the anatomical and diffusion‐weighted sequences did not reveal any pathologies, incidental findings, or spontaneous signal voids. Exemplary color‐coded primary eigenvectors of the diffusion tensor were overlayed on the *b* = 0 s/mm^2^ images acquired with different DTI sequences that are depicted in Figure 2.

### 3.2 Quantitative image analysis

**FIGURE 3:**
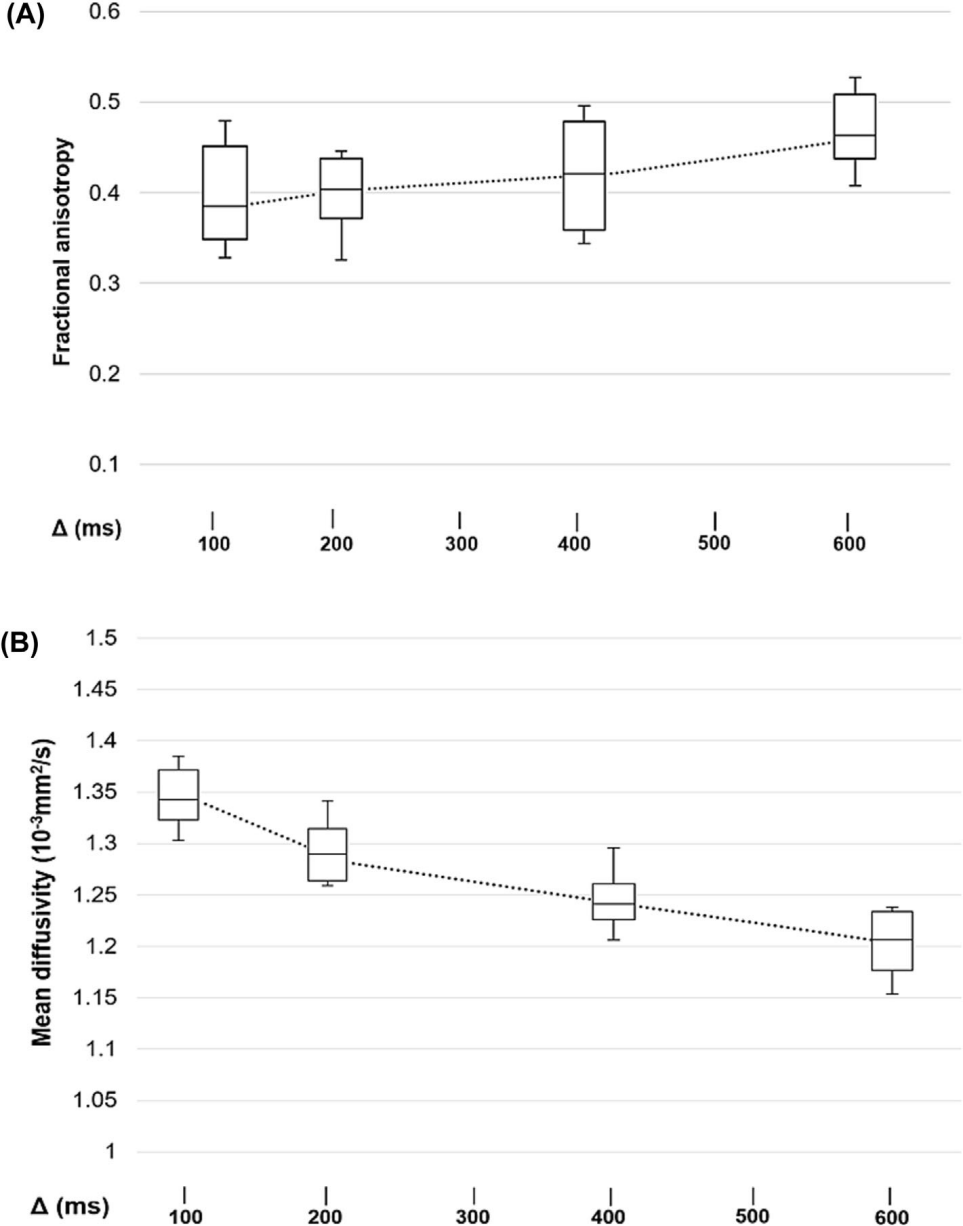
Box plots of fractional anisotropy (FA, A) and mean diffusivity (MD, B) of the STEAM‐DTI sequence in the supraspinatus muscle for Δ of 100/200/400/600 ms. FA continuously increased with longer Δ, whereas MD continuously decreased. DTI, diffusion tensor imaging; STEAM, stimulated echo acquisition mode; Δ, diffusion encoding time*.*

Data obtained with different sequences are summarized in Table 2 and illustrated in Figure 3. FA continuously increased from STEAM at a Δ of 100 ms (hereafter, STEAM_100ms_) (0.38 ± 0.03) to STEAM_600ms_ (0.46 ± 0.02), while it was 0.26 ± 0.02 for SE. Pairwise comparisons revealed a significant difference between SE and STEAM_100ms_ (*p* < 0.001), whereas for STEAM with ascending Δ, no significant difference was found (*p* > 0.999). MD continuously decreased from STEAM_100ms_ (1.33 ± 0.05 [10^−3^ mm^2^/s]) to STEAM_600ms_ (1.20 ± 0.03 [10^−3^ mm^2^/s]), whereas it was 1.62 ± 0.03 (10^−3^ mm^2^/s) for SE. Similar to FA, the difference in MD was significant between SE and STEAM_100ms_ (*p* < 0.001), whereas for STEAM with ascending Δ, no significant difference was found (*p* ≥ 0.340). SNR was significantly higher for SE (72.3 ± 8.7) than for STEAM_100ms–600ms_ (*p* < 0.001, respectively) and continuously decreased with increasing Δ (*p* < 0.001, Table 3).

**TABLE 2:** Diffusion tensor imaging (DTI) parameter values of the supraspinatus muscle derived from SE and STEAM sequences at three different sessions of the study sample (*n* = 10).

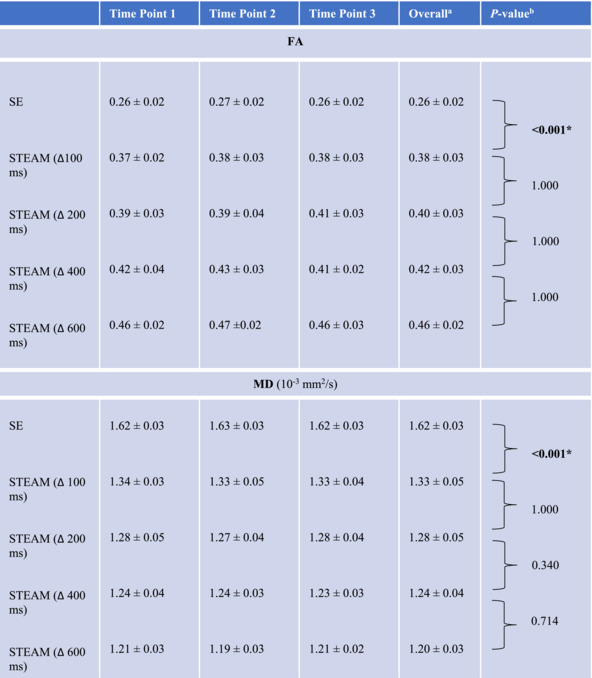

Abbreviations: FA, fractional anisotropy; MD, mean diffusivity; SE, spin echo; STEAM, stimulated echo acquisition mode; Δ, diffusion encoding time.

^a^ Parameter values averaged across all three sessions.

^b^ *P*‐values by ANOVA with Bonferroni‐corrected post‐hoc pairwise comparisons.

* Denotes statistical significance.

**TABLE 3:** Signal‐to‐noise ratio (SNR) of the supraspinatus muscle for different diffusion tensor imaging (DTI) sequences of the study sample (*n* = 10).

	SNR	*P*‐value^a^
SE	72.3 ± 8.7	
STEAM (Δ100 ms)	40.1 ± 4.5	<0.001*
STEAM (Δ200 ms)	37.6 ± 6.2	<0.001*
STEAM (Δ400 ms)	33.2 ± 5.3	<0.001*
STEAM (Δ600 ms)	29.2 ± 7.0	<0.001*

Abbreviations: SE, spin echo; STEAM, stimulated echo acquisition mode.

^a^ *P*‐values by ANOVA with Bonferroni‐corrected post‐hoc pairwise comparisons between SE and STEAM with different Δ.

* Denotes statistical significance.

### 3.3 Test–retest reliability

For FA, all ICCs were found to be excellent (>0.9) for the different DTI sequences (Table 4). SE‐DTI yielded the highest ICC (0.960), while it was lowest for STEAM_100ms_ (0.911). For MD, ICCs were good for SE‐DTI (0.752) and STEAM_100ms–600ms_ (≥0.759). Bland–Altman plots (supporting information Appendix 1) provide an overview of the test–retest reliability of measurements for all DTI sequences between examination sessions 1 and 2, as well as between examination sessions 1 and 3.

**TABLE 4:** Intraclass correlation coefficients (ICCs) between the three examination time points for metrics of different diffusion tensor imaging (DTI) sequences of the study sample (*n* = 10).

	SE‐DTI	STEAM (Δ 100 ms)	STEAM (Δ 200 ms)	STEAM (Δ 400 ms)	STEAM (Δ 600 ms)
FA					
ICC	0.960 (0.872–0.991)	0.920 (0.721–0.967)	0.950 (0.840–0.994)	0.911 (0.846–0.955)	0.933 (0.866–0.969)
MD					
ICC	0.752 (0.556–0.944)	0.810 (0.490–0.889)	0.799 (0.388–0.905)	0.762 (0.585–0.936)	0.759 (0.517–0.910)

*Note:* 95% confidence intervals of all measurements are given in parentheses.

Abbreviations: ICC, intraclass correlation coefficient; FA, fractional anisotropy; MD, mean diffusivity; SE, spin echo; STEAM, stimulated echo acquisition mode; TE, echo time; Δ, diffusion encoding time.

## 4 DISCUSSION

In this study, DTI metrics and SNR in the SSP muscle of healthy individuals were measured with STEAM‐DTI sequences with Δ ranging from 100 to 600 ms and compared with a SE‐DTI sequence.

STEAM resulted in higher FA and lower MD values than SE, while FA increased and MD decreased with increasing Δ. SNR was found to be lower in STEAM compared to SE. The test–retest reliability of FA measurements was found to be good to excellent for both STEAM and SE.

Given that a body of literature exists for STEAM in the leg muscles for the performance of fiber tracking,^7^ assessment of reproducibility^15^ as well as for the assessment of pathological muscle conditions^8^, ^12^, ^16^, ^18^, ^19^ and age‐related microstructural muscle changes,^17^ the aim of this study was to reproduce and transfer previous results to the shoulder muscles. While the values for FA and MD of SE‐DTI were found to be comparable to those observed by Kälin et al,^29^ the parameter change observed in our study can be explained by differences in diffusion times (STEAM: 100–600 ms; SE: ~19 ms).^16^, ^30^ Based on the square root dependence of the free diffusion length on diffusivity and time and assuming a MD of ~1.6 × 10^−3^ mm^2^/s for SE and of ~1.4–1.2 × 10^−3^ mm^2^/s for STEAM as observed in the present study, the corresponding free diffusion length would be 14 and 30–70 μm, respectively.^31^ Consequently, diffusivity would be radially restricted across the sarcolemma membrane during STEAM encoding, but not for SE‐DTI, given that myofiber sizes of the SSP muscle of ~ 25 μm are reported in the current literature.^32^, ^33^ Therefore, the diffusion‐induced phase distributions deviate from a Gaussian shape and are expected to be stretched out along the fiber direction due to the long diffusion time and the lateral confinement by myofibers for STEAM.^13^ Additionally, STEAM provides an image contrast that is more likely to represent diffusion along the myofiber as previously described.^12^ This is achieved by reducing the bias of the signal in muscle from the superimposed signal from fat, thus improving the fidelity of muscle diffusion‐weighted measurements.^7^ As a result, the higher degree of restriction should provide higher sensitivity to pathophysiological structural muscle changes.^16^

SNR was higher for SE‐DTI in our study, which is in line with the current knowledge that STEAM provides ~50% of the signal compared to SE^34^ and can be explained by the signal equations reported by von Deuster et al.^13^ However, the SNR ratio between SE and STEAM is also highly dependent on the TR/TE of the pulse sequences,^7^ as well as the relaxation parameters of the tissue of interest.^35^ Based on the TR/TE of our measurements and T1/T2 of 1420/32 ms for the rotator cuff,^10^ our measured SNR ratio between SE and STEAM with a Δ of 100 ms (180 ± 78%, Table 3), was in very good agreement with the theoretical value (178%)^13^.

Another main finding of this study was that both for STEAM and SE DTI, the test–retest reliability of FA measurements by means of ICC was excellent, despite a long interval (1 week) between the MRI examinations and was better than analogous values reported in previous studies (0.60–0.89).^15^, ^36^, ^37^ As DTI parameters are highly sensitive to temperature and strenuous exercise,^38^, ^39^ this finding might be rationalized by the high standardization of our study setup regarding acclimatization prior to the examination and prohibition of physical activity during the study interval.

On the other hand, the test–retest reliability of MD measurements was slightly higher for STEAM compared to SE but decreased with increasing Δ. While the difference between low and high Δ can be explained by the decreasing SNR, the higher test–retest reliability of STEAM‐DTI compared to SE‐DTI is an interesting finding, particularly in the view of the lower SNR of the STEAM sequence.

The findings regarding test–retest reliability, along with the significant difference in diffusion metrics of STEAM compared to SE, may also be useful for future DTI investigations of muscle disease or changes after exercise, as current studies suggest that extended diffusion times beyond the capabilities of SE may be necessary to depict these pathologies.^34^, ^40^

We acknowledge the following limitations of this study: First, the small sample size may compromise the internal validity of our findings. Second, the analysis of DTI data was performed on 2D ROIs in the anterosuperior muscle bundle, which may have biased our measurements. However, we chose this approach as some authors argue that 2D ROIs are equally reproducible as 3D ROIs.^29^ Third, the shoulder was imaged at an off‐center location at which the *b*‐value is likely to be different from the iso‐center, which tends to be the nominal value selected from the user interface. Fourth, the variability of the low *b*‐value may have resulted in variable perfusion component weighting. To account for this aspect, one could perform a bi‐exponential fit.^41^ Changes in the nominal (high) *b*‐value were comparatively low. This is potentially due to the STEAM sequence which employed a negated polarity of the crusher and slice‐select gradients, such that the net gradient area is minimized during the mixing time.^42^ Additionally, crusher gradients were only applied when diffusion gradients were insufficient to dephase undesired signal pathways. Lastly, it is possible that the transmembrane water exchange may have influenced our measurements, as recently suggested in a study by Malis et al.^17^

In conclusion, this study successfully applied STEAM‐DTI in the supraspinatus muscle of healthy volunteers. Compared to SE‐DTI, STEAM‐DTI exhibited higher FA and lower MD, resulting in an improved diffusion contrast which might be useful for future DTI research. In agreement with theoretical expectations, SNR was lower for STEAM compared to SE. Test–retest reliability of FA measurements was excellent, both for STEAM and SE, whereas the best test–retest reliability of MD measurements was achieved for STEAM at Δ of 100 ms and 200 ms.

Our findings suggest that STEAM can provide radially restricted muscle diffusion without the penalty of reduced test–retest reliability due to insufficient SNR, which might be especially relevant for experiments at higher field strengths (e.g., 7 Tesla). The advocated protocol could be used to study microstructural changes after exercise or muscle disease in the supraspinatus muscle.

## Supporting information


**Appendix S1.** Bland–Altman representation of test–retest reliability for fractional anisotropy (FA; **A, B**) and mean diffusivity (MD; **C, D**) of different DTI acquisitions in sessions 1 and 2 (**A, C**), and in sessions 1 and 3 (**B, D**). *SE, Spin Echo; STEAM, Stimulated Echo Acquisition Mode;* Δ, *Diffusion encoding time*.

**Figure S1.** Supporting Information.

## Data Availability

The data that support the findings of this study are available on request from the corresponding author. The data are not publicly available due to privacy or ethical restrictions.
