# Supplementary material for: Stimulated echo acquisition mode (STEAM) diffusion tensor imaging with different diffusion encoding times in the supraspinatus muscle: Test–retest reliability and comparison to spin echo diffusion tensor imaging
Source: NMR Biomed. 2024 Oct 24;38(1):e5279. doi: 10.1002/nbm.5279 (PMC11602640; doi:10.1002/nbm.5279)
Supplement: Supplementary file 1 — Appendix S1. Bland–Altman representation of test–retest reliability for fractional anisotropy (FA; A, B) and mean diffusivity (MD; C, D) of different DTI acquisitions in sessions 1 and 2 (A, C), and in sessions 1 and 3 (B, D). SE, Spin Echo; STEAM, Stimulated Echo Acquisition Mode; Δ, Diffusion encoding time. [file NBM-38-e5279-s002.docx]

**Supplementary Material**

**Appendix 1.** Bland-Altman representation of test-retest reliability for fractional anisotropy (FA; **A, B**) and mean diffusivity (MD; **C, D**) of different DTI acquisitions in sessions 1 and 2 (**A, C**), and in sessions 1 and 3 (**B, D**). *SE, Spin Echo; STEAM, Stimulated Echo Acquisition Mode;* Δ*, Diffusion encoding time*
