# Supplementary figures and images for: Stimulated echo acquisition mode (STEAM) diffusion tensor imaging with different diffusion encoding times in the supraspinatus muscle: Test–retest reliability and comparison to spin echo diffusion tensor imaging
Source: NMR Biomed. 2024 Oct 24;38(1):e5279. doi: 10.1002/nbm.5279 (PMC11602640; doi:10.1002/nbm.5279)

# SE-DTI

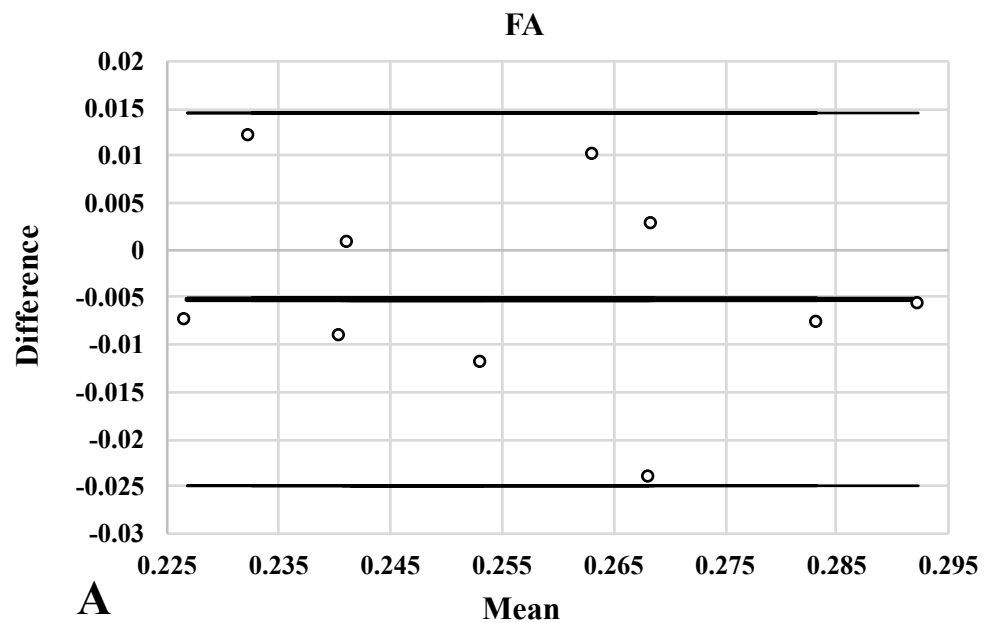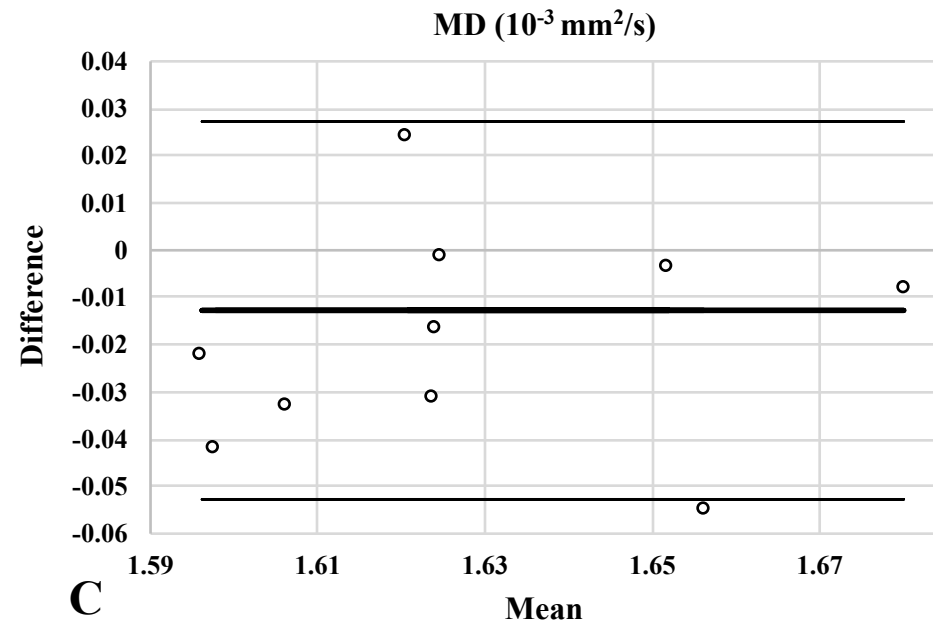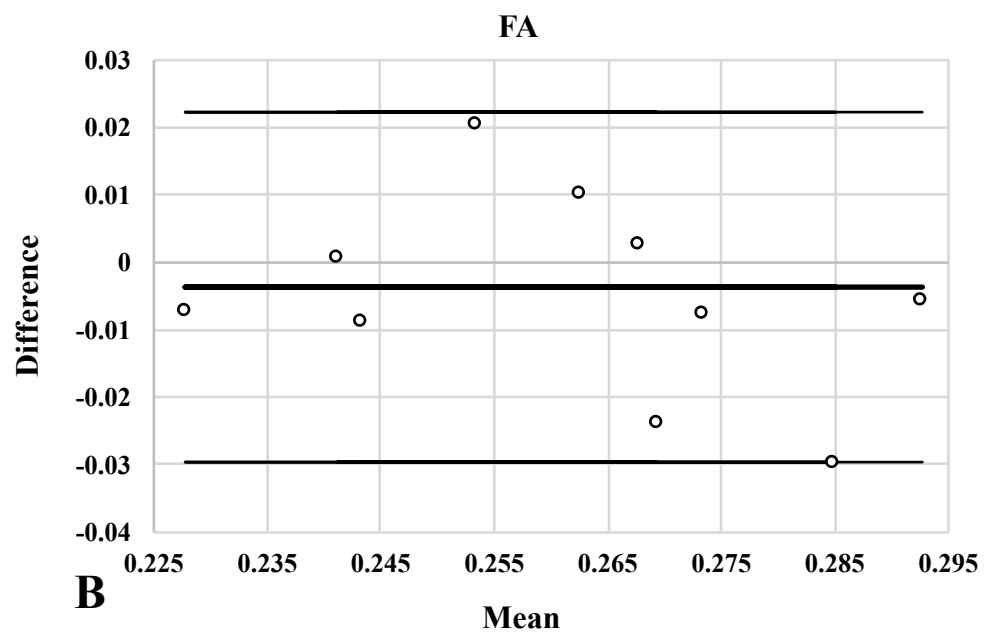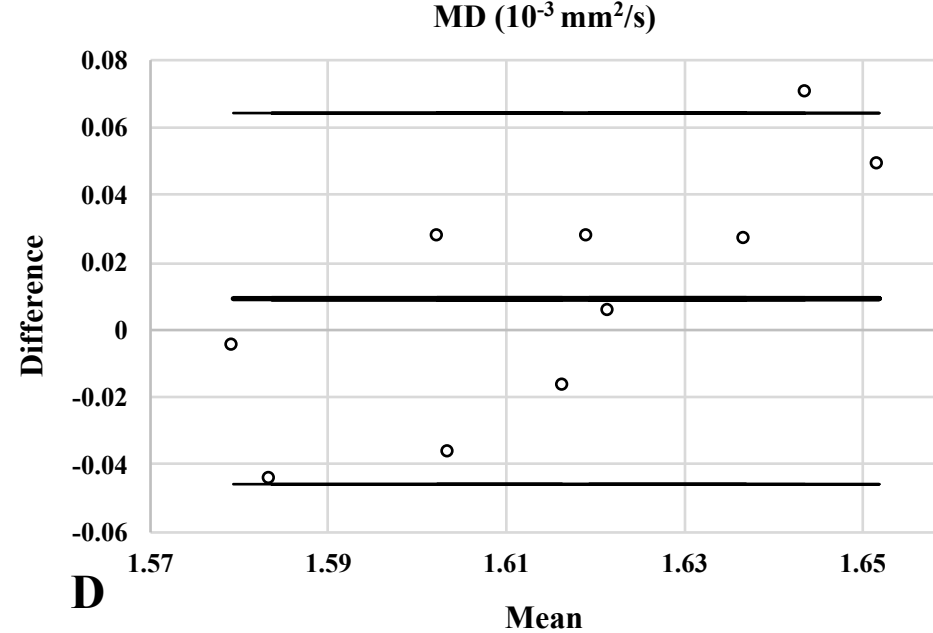

# STEAM ( $\Delta$ 100 ms)

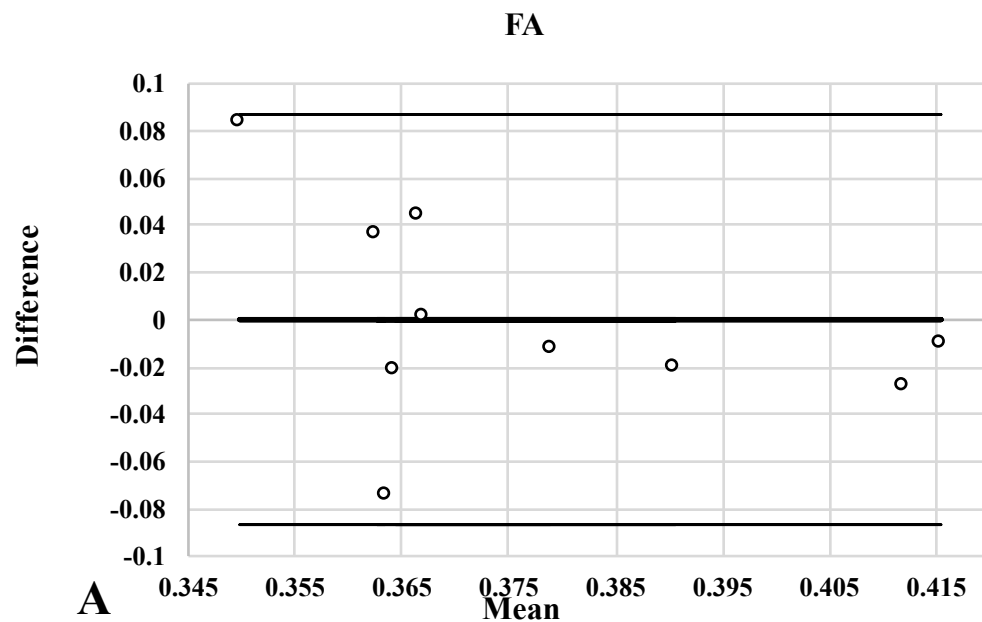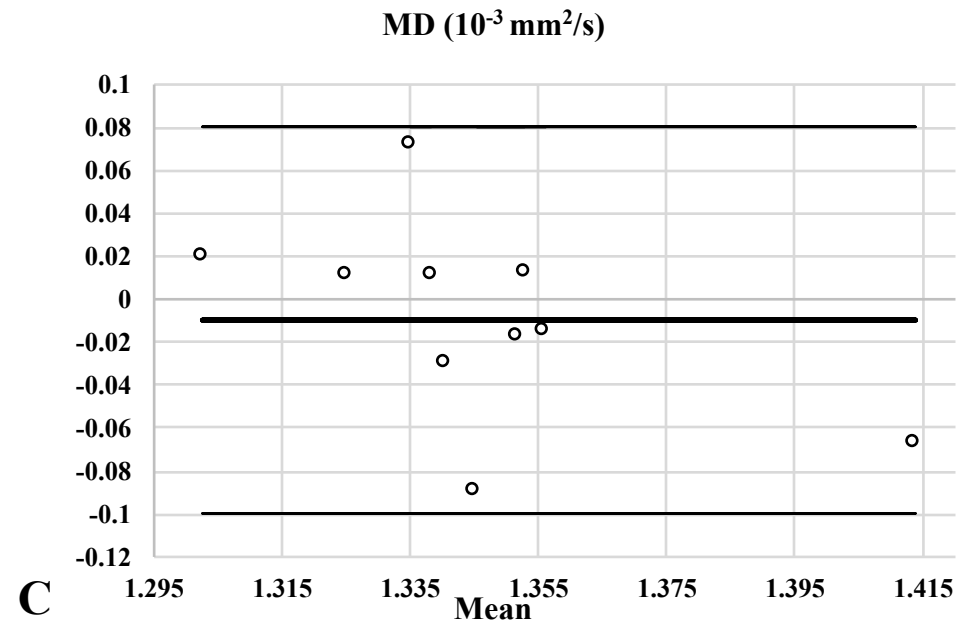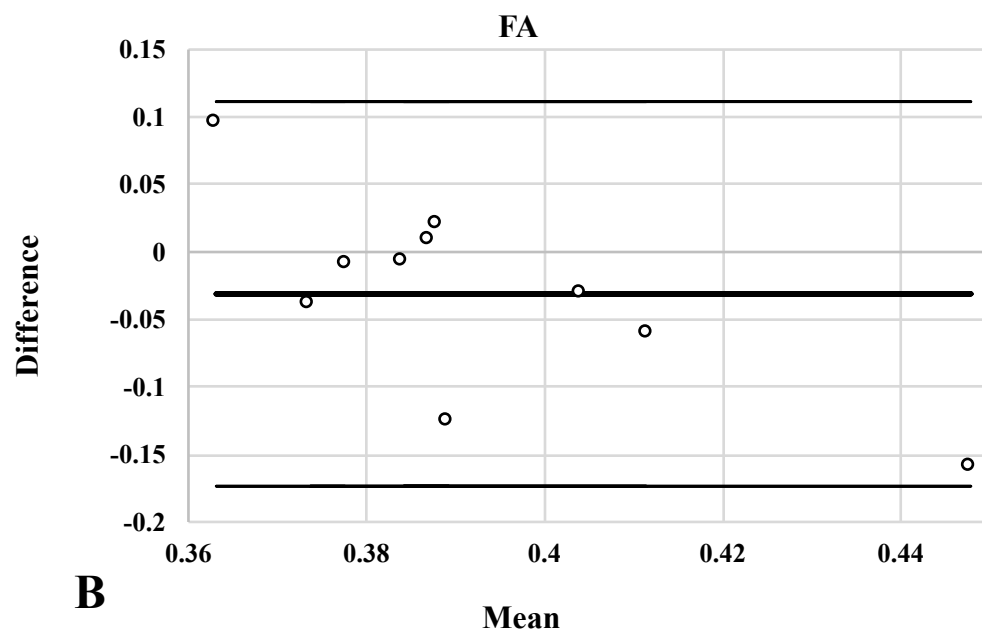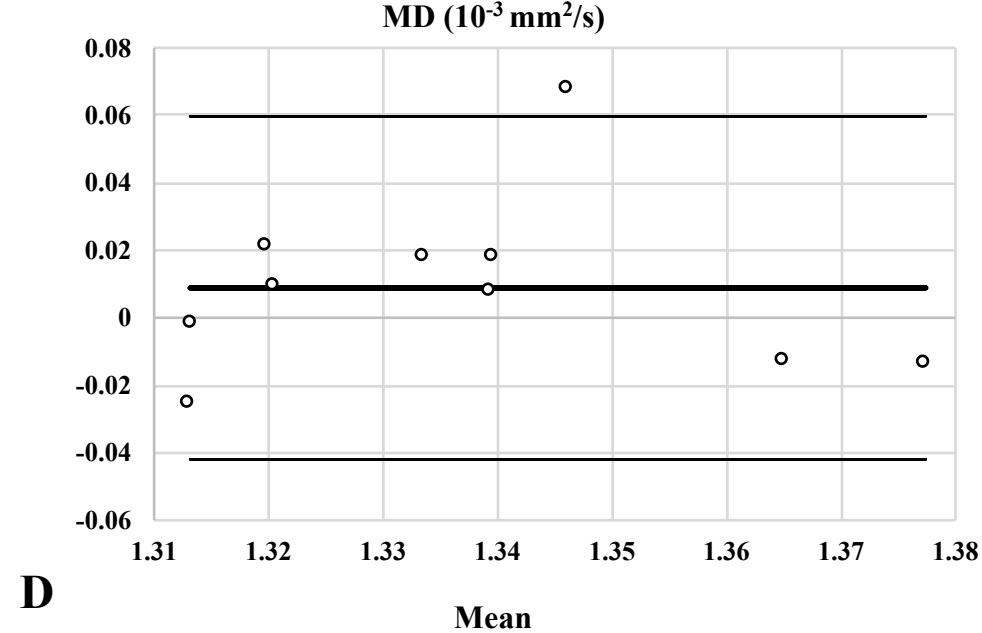

# STEAM ( $\Delta 200$ ms)

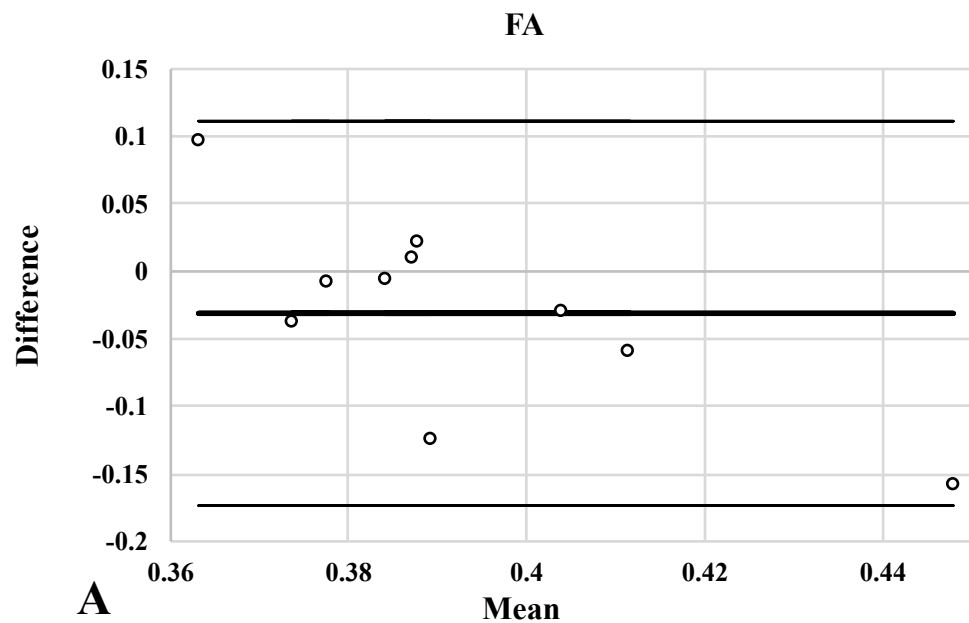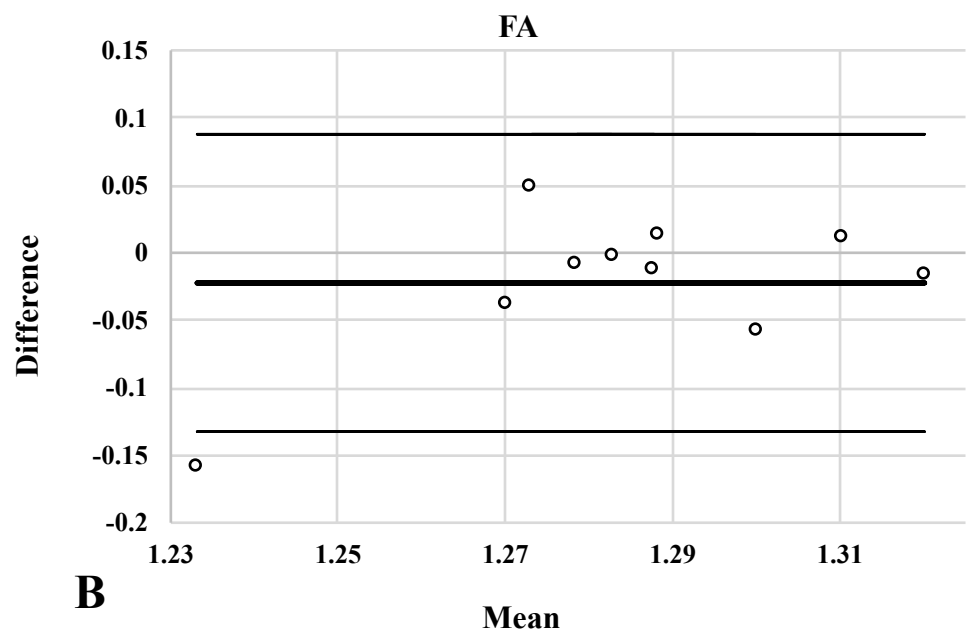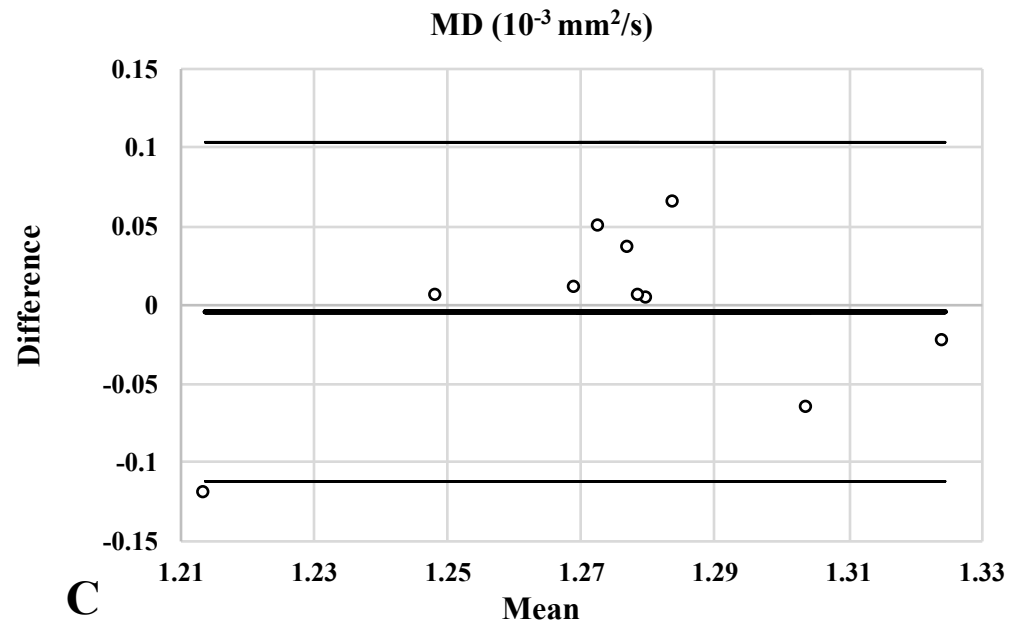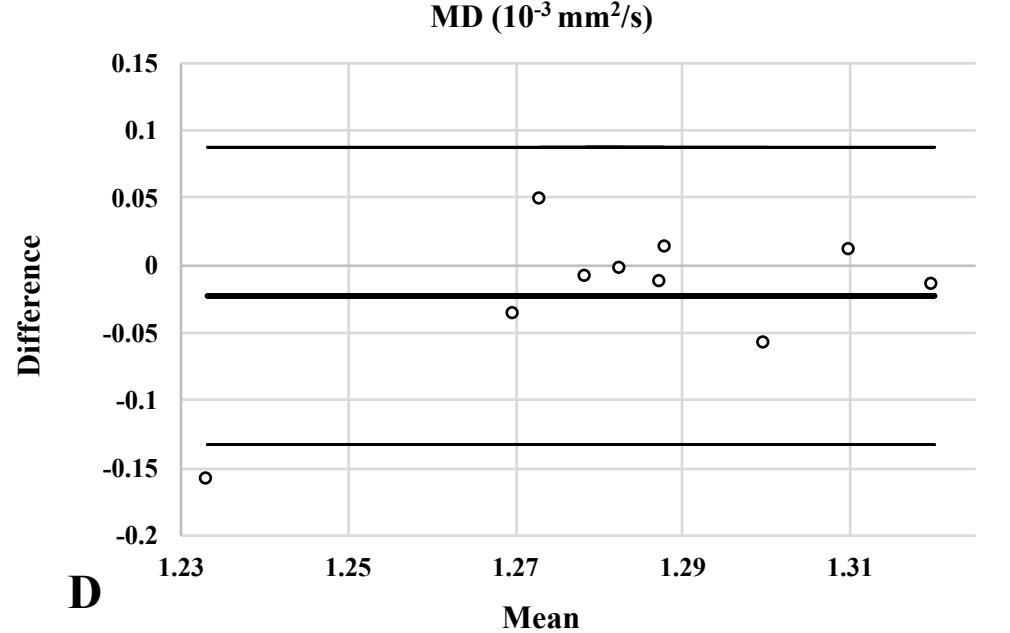

# STEAM ( $\Delta$ 400 ms)

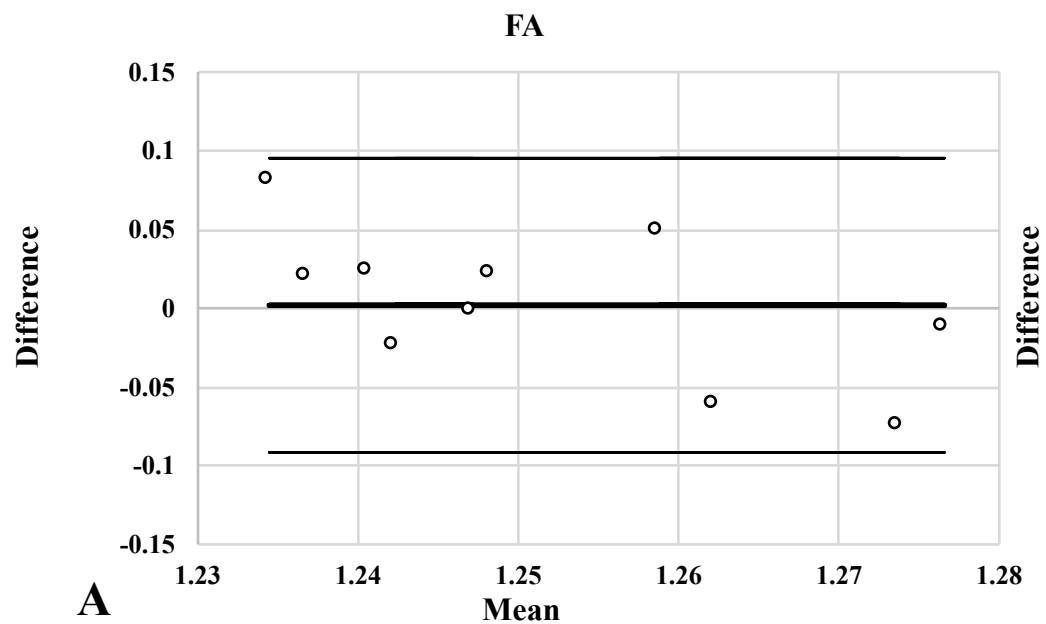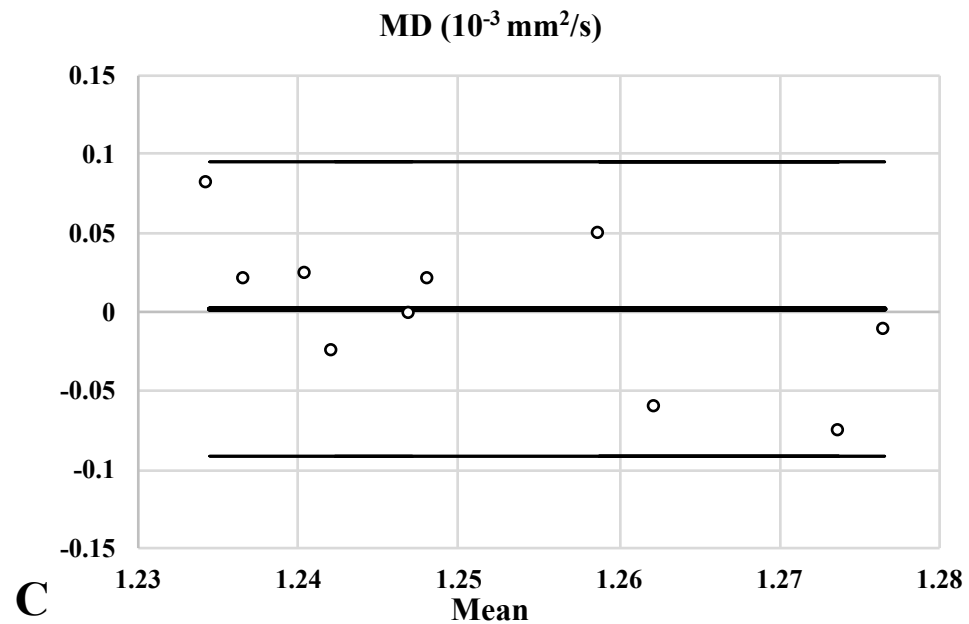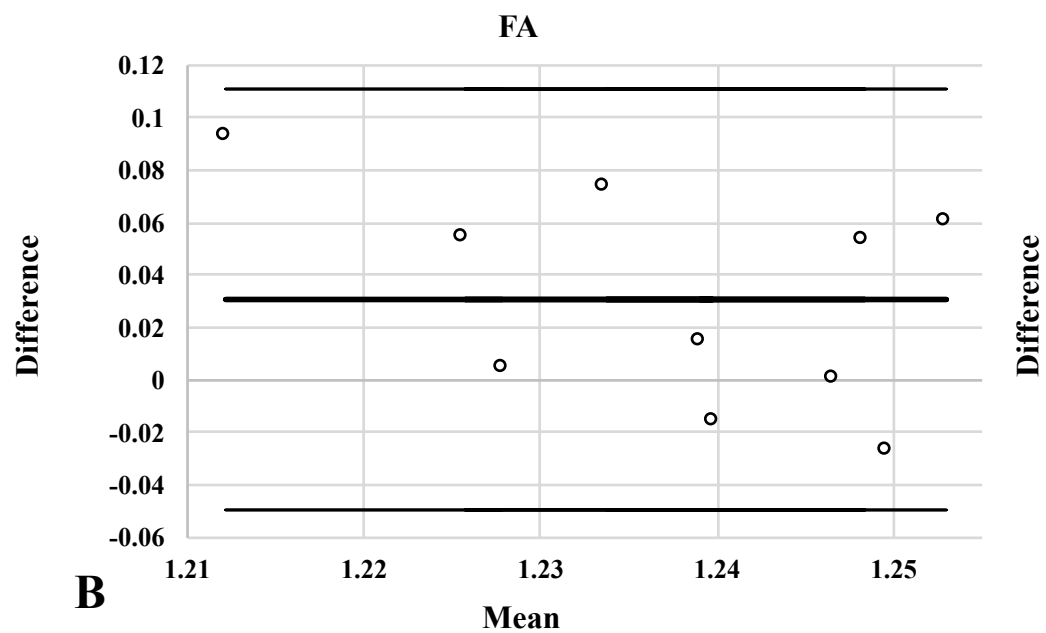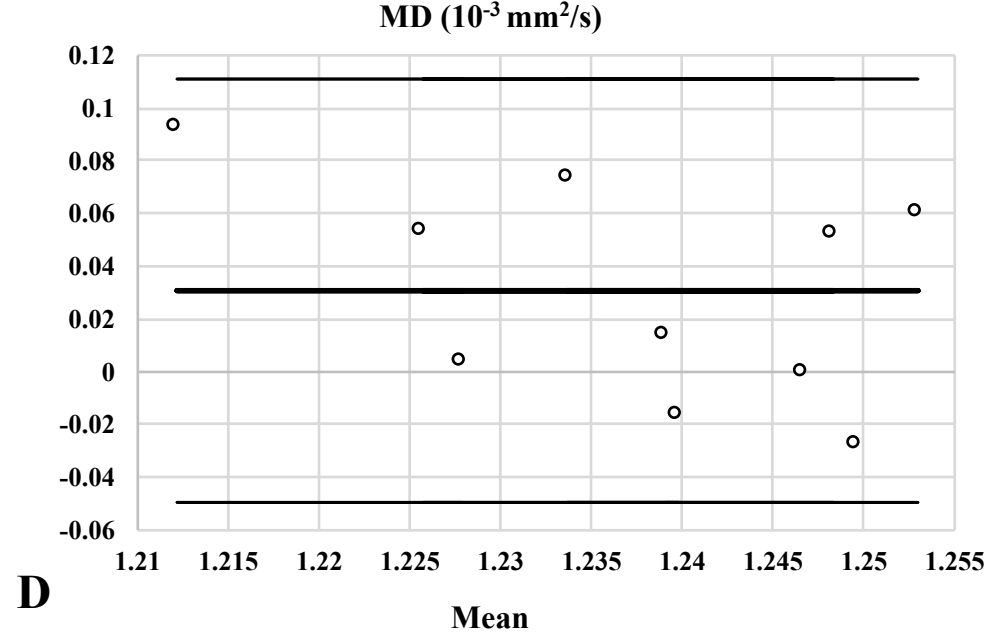

# STEAM ( $\Delta$ 600 ms)

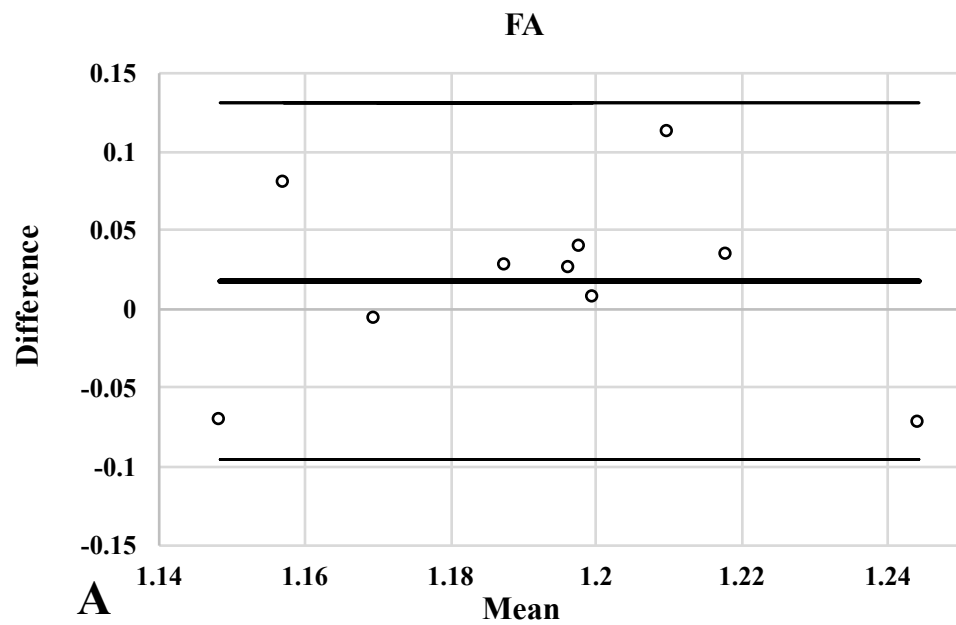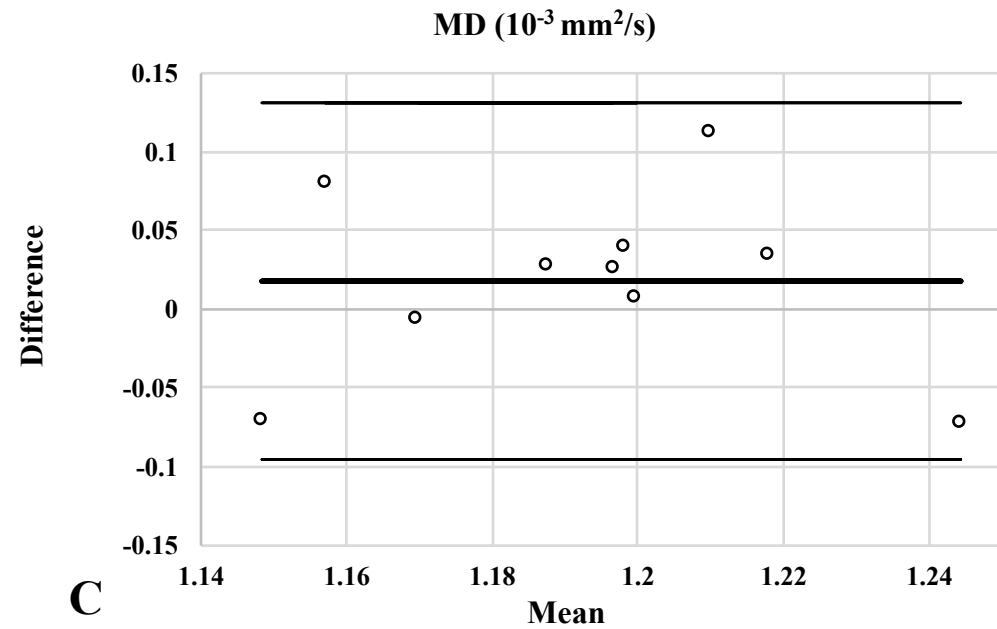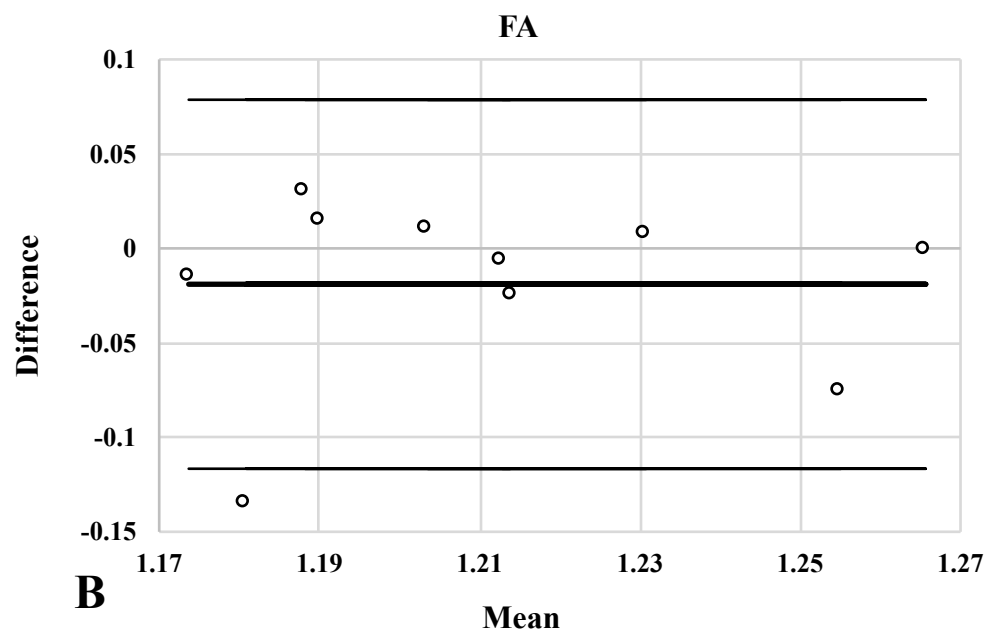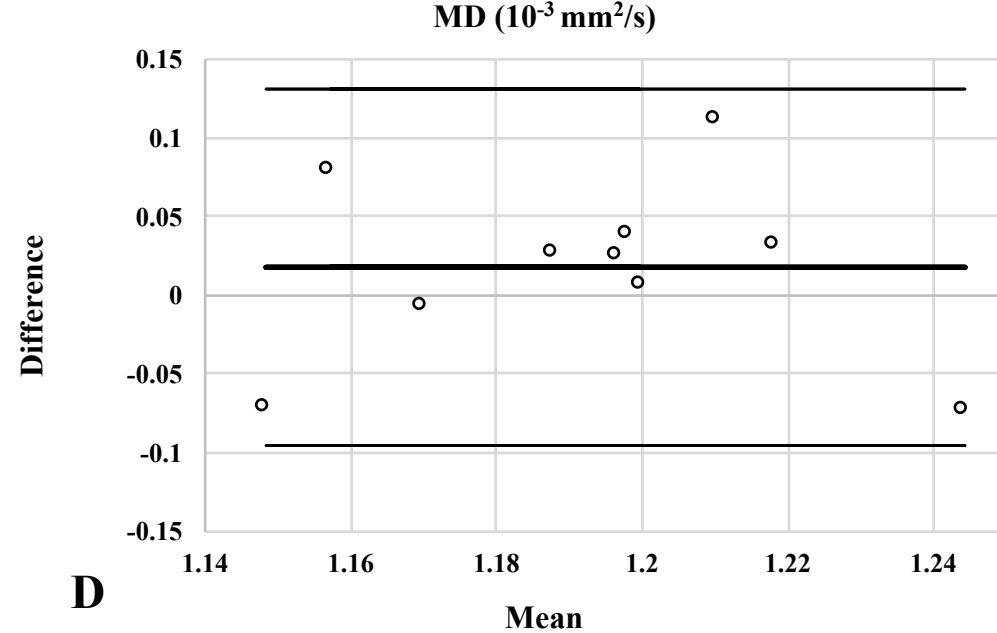

Supplement: Supplementary file 2 — Figure S1. Supporting Information. [file NBM-38-e5279-s001.pdf]
